# Supplementary material for: Iterative improvement in the automatic modular design of robot swarms
Source: PeerJ Comput Sci. 2020 Dec 7;6:e322. doi: 10.7717/peerj-cs.322 (PMC7924708; doi:10.7717/peerj-cs.322)
Supplement: Supplemental Information 3 [file peerj-cs-06-322-s003.zip › argos3/doc/api/standalone/a00482_source.html]

ARGoS: plugins/robots/foot-bot/simulator/pointmass3d\_footbot\_model.cpp Source File


- Main Page
- Related Pages
- Namespaces
- Classes
- Files

- File List
- File Members

# plugins/robots/foot-bot/simulator/pointmass3d\_footbot\_model.cpp

Go to the documentation of this file.

```
00001 
00007 #include "pointmass3d_footbot_model.h"
00008 #include <argos3/core/utility/math/cylinder.h>
00009 
00010 namespace argos {
00011 
00012    static const Real FOOTBOT_RADIUS                   = 0.085036758f;
00013    static const Real FOOTBOT_INTERWHEEL_DISTANCE      = 0.14f;
00014    static const Real FOOTBOT_HEIGHT                   = 0.146899733f;
00015 
00016    enum FOOTBOT_WHEELS {
00017       FOOTBOT_LEFT_WHEEL = 0,
00018       FOOTBOT_RIGHT_WHEEL = 1
00019    };
00020 
00021    /****************************************/
00022    /****************************************/
00023 
00024    CPointMass3DFootBotModel::CPointMass3DFootBotModel(CPointMass3DEngine& c_engine,
00025                                                       CFootBotEntity& c_footbot) :
00026       CPointMass3DModel(c_engine, c_footbot.GetEmbodiedEntity()),
00027       m_cWheeledEntity(c_footbot.GetWheeledEntity()),
00028       m_fCurrentWheelVelocity(m_cWheeledEntity.GetWheelVelocities()) {
00029       /* Register the origin anchor update method */
00030       RegisterAnchorMethod(GetEmbodiedEntity().GetOriginAnchor(),
00031                            &CPointMass3DFootBotModel::UpdateOriginAnchor);
00032       /* Get initial rotation */
00033       CRadians cTmp1, cTmp2;
00034       GetEmbodiedEntity().GetOriginAnchor().Orientation.ToEulerAngles(m_cYaw, cTmp1, cTmp2);
00035    }
00036 
00037    /****************************************/
00038    /****************************************/
00039 
00040    void CPointMass3DFootBotModel::Reset() {
00041       CPointMass3DModel::Reset();
00042       CRadians cTmp1, cTmp2;
00043       GetEmbodiedEntity().GetOriginAnchor().Orientation.ToEulerAngles(m_cYaw, cTmp1, cTmp2);
00044       m_fAngularVelocity = 0.0;
00045    }
00046 
00047    /****************************************/
00048    /****************************************/
00049 
00050 
00051    void CPointMass3DFootBotModel::UpdateFromEntityStatus() {
00052       m_cVelocity.Set((m_fCurrentWheelVelocity[FOOTBOT_RIGHT_WHEEL] + m_fCurrentWheelVelocity[FOOTBOT_LEFT_WHEEL])*0.5, 0.0, 0.0);
00053       m_cVelocity.RotateZ(m_cYaw);
00054       m_fAngularVelocity = (m_fCurrentWheelVelocity[FOOTBOT_RIGHT_WHEEL] - m_fCurrentWheelVelocity[FOOTBOT_LEFT_WHEEL]) / FOOTBOT_INTERWHEEL_DISTANCE;
00055    }
00056 
00057    /****************************************/
00058    /****************************************/
00059 
00060    void CPointMass3DFootBotModel::Step() {
00061       m_cPosition += m_cVelocity * m_cPM3DEngine.GetPhysicsClockTick();
00062       m_cYaw += CRadians(m_fAngularVelocity * m_cPM3DEngine.GetPhysicsClockTick());
00063    }
00064 
00065    /****************************************/
00066    /****************************************/
00067 
00068    void CPointMass3DFootBotModel::CalculateBoundingBox() {
00069       GetBoundingBox().MinCorner.Set(
00070          GetEmbodiedEntity().GetOriginAnchor().Position.GetX() - FOOTBOT_RADIUS,
00071          GetEmbodiedEntity().GetOriginAnchor().Position.GetY() - FOOTBOT_RADIUS,
00072          GetEmbodiedEntity().GetOriginAnchor().Position.GetZ());
00073       GetBoundingBox().MaxCorner.Set(
00074          GetEmbodiedEntity().GetOriginAnchor().Position.GetX() + FOOTBOT_RADIUS,
00075          GetEmbodiedEntity().GetOriginAnchor().Position.GetY() + FOOTBOT_RADIUS,
00076          GetEmbodiedEntity().GetOriginAnchor().Position.GetZ() + FOOTBOT_HEIGHT);
00077    }
00078 
00079    /****************************************/
00080    /****************************************/
00081 
00082    bool CPointMass3DFootBotModel::CheckIntersectionWithRay(Real& f_t_on_ray,
00083                                                            const CRay3& c_ray) const {
00084       CCylinder m_cShape(FOOTBOT_RADIUS,
00085                          FOOTBOT_HEIGHT,
00086                          m_cPosition,
00087                          CVector3::Z);
00088       bool bIntersects = m_cShape.Intersects(f_t_on_ray, c_ray);
00089       return bIntersects;
00090    }
00091 
00092    /****************************************/
00093    /****************************************/
00094 
00095    void CPointMass3DFootBotModel::UpdateOriginAnchor(SAnchor& s_anchor) {
00096       s_anchor.Position = m_cPosition;
00097       s_anchor.Orientation = CQuaternion(m_cYaw, CVector3::Z);
00098    }
00099 
00100    /****************************************/
00101    /****************************************/
00102    
00103    REGISTER_STANDARD_POINTMASS3D_OPERATIONS_ON_ENTITY(CFootBotEntity, CPointMass3DFootBotModel);
00104 
00105    /****************************************/
00106    /****************************************/
00107 
00108 }
```

---

Generated on 10 Jul 2018 for ARGoS by 
 1.6.1
